# Geographic Variation in Obesity at the State Level in the All of Us Research Program

**DOI:** 10.5888/pcd18.210094

**Published:** 2021-12-23

**Authors:** Cheryl R. Clark, Paulette D. Chandler, Guohai Zhou, Nyia Noel, Confidence Achilike, Lizette Mendez, George T. O’Connor, Jordan W. Smoller, Scott T. Weiss, Shawn N. Murphy, Mark J. Ommerborn, Jason H. Karnes, Yann C. Klimentidis, Christina D. Jordan, Robert A. Hiatt, Andrea H. Ramirez, Roxana Loperena, Kelsey Mayo, Elizabeth Cohn, Lucila Ohno-Machado, Eric Boerwinkle, Mine Cicek, Sheri D. Schully, Stephen Mockrin, Kelly A. Gebo, Elizabeth W. Karlson

**Affiliations:** 1 Department of Medicine, Brigham and Women’s Hospital, Harvard Medical School, Boston, Massachusetts; 2 Department of Obstetrics and Gynecology, Boston Medical Center, Boston University School of Medicine, Boston, Massachusetts; 3 Pulmonary Center, Boston Medical Center, Boston University School of Medicine, Boston, Massachusetts; 4 Center for Genomic Medicine, Massachusetts General Hospital, Boston, Massachusetts; 5 Channing Division of Network Medicine, Department of Medicine, Brigham and Women’s Hospital, Boston, Massachusetts; 6 Research Information Science and Computing, Mass General Brigham, Boston, Massachusetts; 7 Department of Pharmacy Practice and Science, University of Arizona College of Pharmacy, Tucson, Arizona; 8 Department of Epidemiology and Biostatistics, Mel and Enid Zuckerman College of Public Health, University of Arizona, Tucson, Arizona; 9 University of Mississippi Medical Center, Jackson, Mississippi; 10 Department of Epidemiology and Biostatistics, University of California, San Francisco, California; 11 Department of Medicine, Vanderbilt University Medical Center, Nashville, Tennessee; 12 All of Us Research Program, National Institutes of Health, Bethesda, Maryland; 13 Medical Affairs, Inflammation and Autoimmunity, Incyte Corporation, Wilmington, Delaware; 14 Vanderbilt Institute for Clinical and Translational Research, Vanderbilt University Medical Center, Nashville, Tennessee; 15 Hunter-Bellevue School of Nursing, Hunter College, City University of New York, New York, New York; 16 Department of Biomedical Informatics, University of California San Diego Health, La Jolla, California; 17 School of Public Health, The University of Texas Health Science Center at Houston, Houston, Texas; 18 Department of Laboratory Medicine and Pathology, Mayo Clinic, Rochester, Minnesota; 19 Life Sciences Division, Leidos, Inc, Frederick, Maryland; 20 Department of Medicine, Johns Hopkins University School of Medicine, Baltimore, Maryland

## Abstract

**Introduction:**

National obesity prevention strategies may benefit from precision health approaches involving diverse participants in population health studies. We used cohort data from the National Institutes of Health All of Us Research Program (All of Us) Researcher Workbench to estimate population-level obesity prevalence.

**Methods:**

To estimate state-level obesity prevalence we used data from physical measurements made during All of Us enrollment visits and data from participant electronic health records (EHRs) where available. Prevalence estimates were calculated and mapped by state for 2 categories of body mass index (BMI) (kg/m^2^): obesity (BMI >30) and severe obesity (BMI >35). We calculated and mapped prevalence by state, excluding states with fewer than 100 All of Us participants.

**Results:**

Data on height and weight were available for 244,504 All of Us participants from 33 states, and corresponding EHR data were available for 88,840 of these participants. The median and IQR of BMI taken from physical measurements data was 28.4 (24.4– 33.7) and 28.5 (24.5–33.6) from EHR data, where available. Overall obesity prevalence based on physical measurements data was 41.5% (95% CI, 41.3%–41.7%); prevalence of severe obesity was 20.7% (95% CI, 20.6–20.9), with large geographic variations observed across states. Prevalence estimates from states with greater numbers of All of Us participants were more similar to national population-based estimates than states with fewer participants.

**Conclusion:**

All of Us participants had a high prevalence of obesity, with state-level geographic variation mirroring national trends. The diversity among All of Us participants may support future investigations on obesity prevention and treatment in diverse populations.

**Summary**

**What is already known on this topic?**

The prevalence of obesity in the US varies by geographic location and personal characteristics. Data on individual and population-level characteristics in diverse populations can benefit research.

**What is added by this report?**

We used All of Us data to examine the prevalence of obesity at the state level and tested its validity by comparing results to an existing large-scale data source.

**What are the implications for public health practice?**

Data from the All of Us Research Program may have sufficient geographic and demographic diversity for population-level studies of obesity.

## Introduction

Efforts to address the growing obesity epidemic in the US may benefit from precision health approaches that use integrated data on environments, social determinants of health, health behaviors, clinical conditions, and genomic factors that contribute to risks in individuals and in diverse populations (1,2). Few cohorts have sufficient size or diversity of data types and populations needed to investigate the multiple potential contributors to obesity in the US. The National Institutes of Health’s (NIH’s) All of Us Research Program (All of Us) is designed to integrate multiple data types for research, with the goal of including data from 1 million people collected longitudinally over 10 years. Baseline assessments include in-person study visits, during which physical measurements are taken by trained study staff, including height and weight measurements (3). Clinical data, including height and weight measurements used during clinical encounters, are also collected from electronic health records (EHRs) of All of Us participants who consent to provide these data. Reports of behavioral, environmental, social, and demographic characteristics are collected through surveys administered to a diverse participant population. Biologic samples, including blood samples obtained via venipuncture, are obtained for biomarker and genomic studies.

Population-based data, such as results of the Behavioral Risk Factor Surveillance System (BRFSS), are available to study obesity in the US. BRFSS is a large, nationally representative, telephone-based survey of more than 400,000 participants conducted annually by state health departments to collect information on self-reported risk behaviors, chronic health conditions, and use of prevention services (4). Additionally, the National Health and Nutrition Examination Survey (NHANES) captures data annually on a nationally representative sample of approximately 5,000 participants and includes data from survey interviews, in-person physical measurements, and laboratory tests (5). BRFSS and NHANES have relative strengths and limitations for conducting obesity research at the population level. The large size of BRFSS enables monitoring of population-based obesity prevalence at the state level. However, measures of weight and height in BRFSS are obtained by self-report and may be subject to underestimating obesity because of self-reporting bias (1). The smaller NHANES study data are collected in an examination unit and thus provide objective physical measurements rather than self-reported data. However, NHANES data are designed to provide estimates that are nationally representative, but not representative of smaller geographic areas (1). To address these concerns, Ward et al generated state-level projections of obesity prevalence in BRFSS data that correct for self-reporting bias by using the distribution of obesity in NHANES as a correction factor (1). Data from All of Us may contribute additional value to these existing population-based resources because of the large size and nationwide distribution of the All of Us cohort for which objective measurements and biomarker data are available through in-person measurement, along with longitudinal data collected through EHRs that are not available in other cohort studies of this scale. Additionally, participant diversity within All of Us may provide insight into factors relevant to obesity risk in various social and geographic contexts and population strata in the US. More than 80% of All of Us participants belong to population groups that have been historically underrepresented in biomedical research, including people who are aged 65 or older, Black or Hispanic, have low income (annual income below the federal poverty level), less than a high school diploma or equivalent, diverse sexual orientation and gender identities, and rural residents (3).

To facilitate research, the All of Us Researcher Workbench was developed to provide access to integrated data types in the program. All of Us cohort data types include physical measurements, EHR data, surveys, and biospecimens. Data on height and weight from EHR and physical measurements data sources have not previously been reported. The goal of our study was to demonstrate the utility of the All of Us Researcher Workbench for examining obesity prevalence across the US in the All of Us cohort. First, our study validated the data on height and weight by examining the concordance of the 2 data sources for height and weight (physical measurements and EHR) in All of Us. Second, the study estimated state-level obesity prevalence among All of Us participants by sex in physical measurements data, and compared and contrasted our estimates with BRFSS data previously reported at the state level by Ward et al (1).

## Methods

**All of Us demonstration projects**. We conducted our study from May 2018 through December 2020 (data collected in this range are date stamped March 8, 2021). The goals, recruitment methods, study sites, and scientific rationale for All of Us have been described previously (6). Demonstration projects were designed to describe the All of Us cohort and reproduce previous studies for validation purposes. Our study was proposed by members of the All of Us investigator consortium and reviewed and overseen by the program’s science committee. Our analysis of deidentified data was classified as research not involving human subjects by the All of Us institutional review board. The initial release of data and tools used in our study was published recently (7). Results reported are in compliance with the All of Us Data and Statistics Dissemination Policy disallowing disclosure of results in group counts under 20.

**All of Us Researcher Workbench**. Our study used data available through the All of Us Researcher Workbench, a cloud-based platform where approved researchers can access and analyze All of Us data (6). The details of the surveys and methods of data collection are available in the Survey Explorer found in the All of Us Research Hub (https://www.researchallofus.org), a website designed to support researchers (8). Three currently available data types (survey, physical measurements, and EHR) are mapped to the common data model of the Observational Medical Outcomes Partnership, version 5.2, maintained by the Observational Health Data Sciences and Informatics collaborative (https://www.ohdsi.org/data-standardization). To protect participant privacy, a series of data transformations were applied. These included data suppression of codes with a high risk of identification, such as military status; generalization of categories, including age, sex at birth, gender identity, sexual orientation, and race or ethnicity; and date shifting by a random (less than 1 year) number of days, implemented consistently across each participant record. Documentation on privacy implementation and creation of a curated data repository (CDR) is available in the All of Us Registered Tier CDR Data Dictionary (9). The Researcher Workbench currently offers tools with a user interface built for selecting groups of participants, creating data sets for analysis, and workspaces (Jupyter Notebooks; https://www.jupyter.org) to analyze data. The Notebooks enable the use of saved data sets and direct query by using R (R Project for Statistical Computing) and Python 3 (Python) programming languages. This demonstration project used the All of Us curated data set (CDR version fc-aou-cdr-prod.R2020Q4R2) on a secure server on March 8, 2021, by using a Researcher Workbench interface, version 4, which includes data released by the program in December 2020.

**Study population**. Enrollment in All of Us began in May 2018, and the program currently enrolls participants aged 18 or older from a network of recruitment sites in more than 41 states. Enrollment will continue until at least 1 million participants are enrolled (3). All of Us is designed to recruit people who are underrepresented in biomedical research with the goal of enrolling its cohort from populations that are more than 75% underrepresented in terms of demographics, geographic location, and other characteristics, with at least 45% of participants coming from racial and ethnic groups that are underrepresented in research (3). Information on the sites from which participants are recruited has been described (6). Briefly, recruitment sites for All of Us were selected via an NIH submission and review process. The number of recruitment sites is evolving, and as of this writing, All of Us participants were enrolled at regional medical centers (93.6%), federally qualified health centers (3.2%), Veterans Health Administration sites (1.6%), and “direct volunteer” sites that can provide access for people who are not patients in a health care organization (a designated health clinic, blood bank, laboratory, or other facility) (1.6%). Participants in All of Us enroll digitally and provide informed consent to participate in the program through the website (https://www.joinallofus.org), via a smartphone application, or through one of the participating recruitment sites. After a person 1) consents to participate, 2) provides authorization to share EHR data, or 3) completes the initial baseline survey of demographic information, the participant becomes eligible for in-person visits to have physical measurements and biospecimens collected at one of the All of Us recruitment sites.

**Data collection from in-person physical measurements and EHRs.** Study protocols at each recruitment site were followed to measure objective height and weight during in-person visits. Height is measured via stadiometer and recorded in centimeters to the nearest millimeter. Weight is recorded in kilograms to the nearest 0.1 kg. Clinical data on height, weight, and calculated body mass index (BMI) (weight in kg/height in m^2^) that were collected and recorded in participant EHRs during in-person clinical visits for routine patient care were extracted and transformed into the Observational Medical Outcomes Partnership common format at each enrollment site (7). For our analysis, height and weight values from physical measurements visits and EHR data were used to calculate BMI from both data sources. Our analyses also included survey data on demographics (sex and gender identity, education, race or ethnicity, age, and geographic location as US state of residence).

**Statistical methods**. We used the methods of Ward et al (1) to examine 2 categories of obesity consistent with Centers for Disease Control and Prevention (CDC) definitions of overall obesity (BMI ≥30) and severe obesity (BMI ≥35). We calculated obesity categories separately from physical measurements data only and EHR data only. Among those with both sources of data, we examined the correlation between the two with Pearson correlation coefficients. We examined baseline characteristics of participants from physical measurements data only and those who also contributed EHR data. We compared the characteristics of those with and without EHR data with χ^2^ tests of significance for categorical variables. Sufficient data were available to display state-level obesity estimates by using All of Us BMI measurements calculated from physical measurements data, but at this writing, a sufficient sample of EHR data currently collected by All of Us was unavailable to display state-level estimates. We calculated the prevalence of obesity and severe obesity nationwide and for each state, overall and separately for men and women. We also calculated the prevalence of obesity or severe obesity in a complete-case analysis for All of Us participants with known data on age, binary sex assigned at birth (male or female), race or ethnicity (Black, White, Hispanic, and other groups), height and weight, and education levels (<high school diploma or equivalent, high school diploma to some college, college graduate). Data from participants with nonbinary gender identities were not reported in these results because of small sample sizes. After deletion of participants with incomplete data, we compared the prevalence of obesity and severe obesity to reported BRFSS projections adjusted for self-report bias by Ward et al (1). We used ArcGIS version 10.7.1 (Esri) to map physical measurements of BMI data by state.

**Exclusions and missing data**. We excluded 9 states (Idaho, Iowa, Kentucky, Maine, Montana, New Hampshire, Ohio, Oklahoma, and Virginia) and the District of Columbia because they had fewer than 100 participants with height and weight data from physical measurements. We excluded individuals for whom information on the state of residence was not available or was suppressed to protect privacy. We included a total of 33 states (Alabama, Arizona, Arkansas, California, Colorado, Connecticut, Florida, Georgia, Illinois, Indiana, Kansas, Louisiana, Maryland, Massachusetts, Michigan, Minnesota, Mississippi, Missouri, Nevada, Nebraska, New Jersey, New Mexico, New York, North Carolina, North Dakota, Oregon, Pennsylvania, South Carolina, Tennessee, Texas, Utah, Washington, and Wisconsin) in the analysis. Other states and territories either had no All of Us participants or had participant information suppressed because of low participant numbers.

To select height measurements from EHR data, we used Standard Concept Names (body height, body height measured, or body height stated), or the Source Concept Names (height, body height, or body height stated). To select weight measurements, we used Standard Concept Names (body weight, dry body weight measured, body weight measured, or body weight stated) or Source Concept names (weight, body weight, or body weight stated) (10). As quality control of EHR height and weight measurements, we used the methods of Koebnick et al (11) describing a large young adult multiethnic cohort to guide our weight, height, and BMI exclusion criteria. We excluded inpatient and emergency department visits, visits during pregnancy, body weight below 30 lb or above 1,000 lb, height below 4 ft or above 7 ft, 2 in, and BMI <5 kg/m^2^ or ≥100 kg/m^2^ (11). These exclusion and inclusion criteria were used for both physical measurements and measurements taken from EHR data sources (11). For people with multiple physical measurements of height or weight, the average measurement was taken (to reduce measurement error) and the most recent physical measurement date was used. Only EHR height and weight measurements taken within 1 year of physical measurements were used. For participants with multiple EHR height and weight measurements, we took the EHR height/weight measurement on the date closest to the physical measurements visit date. We also excluded 166 participants whose weight numbers from physical measurements consistently deviated from their EHR weight numbers, suggesting a probable documentation error in physical measurements weight units. Data were analyzed in the All of Us Research Workbench Jupyter notebook by using R software version 4.0.2.

## Results

Physical measurements data were available for 244,504 participants (Table 1). The mean age of participants with physical measurements data in this study was 51.1 years. EHR data were available from 88,840 (36.3%) study participants with physical measurements data, with a mean age of 53.9 years. The overall median and IQR of BMI using physical measurements data was 28.4 (24.4–33.7). The overall median and IQR of BMI using EHR data was 28.5 (24.5–33.6). Participants who contributed only physical measurements data and not EHR data were more likely to be male and underrepresented in biomedical research, including 26.3% who were non-Hispanic Black, 23.2% who were Hispanic, 13.6% who did not have a high school diploma or equivalent, and 50.4% who had a high school diploma or equivalent (Table 1). EHR data were less frequently available from study participants from states in the South and West/Pacific than other regions.

**Table 1:** Physical Measurements and Electronic Health Records Data^a^, Participants With Binary Sex Assigned at Birth, by Demographic Characteristics and US Region, All of Us Research Program, May 2018–December 2020

Subgroup	Total Cohort, n (%) (N = 244,504)	Cohort Participants Contributing Physical Measurements Data Only, n (%) (n = 155,664)	Cohort Participants Contributing Physical Measurements and EHR Data, n (%) (n = 88,840)	*P* Value
**Binary sex assigned at birth**				
Male	95,944 (39.2)	63,294 (40.7)	32,650 (36.8)	<.001
Female	148,560 (60.8)	92,370 (59.3)	56,190 (63.2)	
**Race or ethnicity**				
Non-Hispanic White	120,519 (49.3)	68,085 (43.7)	52,434 (59.0)	<.001
Non-Hispanic Black	57,341 (23.5)	40,877 (26.3)	16,464 (18.5)	
Hispanic	50,889 (20.8)	36,064 (23.2)	14,825 (16.7)	
Other race or ethnicity^b^	15,755 (6.4)	10,638 (6.8)	5,117 (5.8)	
**Education**				
Less than high school diploma or equivalent	28,709 (11.7)	21,103 (13.6)	7,606 (8.6)	<.001
High school diploma or equivalent to some college	118,906 (48.6)	78,458 (50.4)	40,448 (45.5)	
College graduate	96,889 (39.6)	56,103 (36.0)	40,786 (45.9)	
**Age, y**				
18–39	72,110 (29.5)	50,512 (32.4)	21,598 (24.3)	<.001
40–64	114,472 (46.8)	73,601 (47.3)	40,871 (46.0)	
≥65	57,922 (23.7)	31,551 (20.3)	26,371 (29.7)	
**Region**				
**Northeast**				
Massachusetts, Connecticut, New Jersey, New York, Pennsylvania	69,309 (28.3)	34,518 (22.2)	34,791 (39.2)	<.001
**South**				
Alabama, Florida, Georgia, Louisiana, Mississippi, South Carolina, Tennessee, Texas	50,806 (20.8)	38,046 (24.4)	12,760 (14.4)	<.001
**Midwest**				
Illinois, Michigan, Minnesota, Wisconsin	48,881 (20.0)	26,934 (17.3)	21,947 (24.7)	<.001
**West and Pacific**				
Arizona, California, Colorado, Nevada, New Mexico	66,785 (27.3)	51,730 (33.2)	15,055 (16.9)	<.001
**Region unknown**				
State information suppressed for privacy reasons	8,723 (3.6)	4,436 (2.8)	4,287 (4.8)	<.001

Abbreviation: EHR, electronic health record.

a Physical measurements and data are from All of Us participants with known age, binary sex, race, and education and from states with at least 100 participants. Percentages may not sum to 100% because of rounding.

b Participants for whom data are not reported separately because of small sample sizes. These include those responding none of these, Asian, more than one race or ethnicity, or another single race or ethnicity.

**Figure 1:**
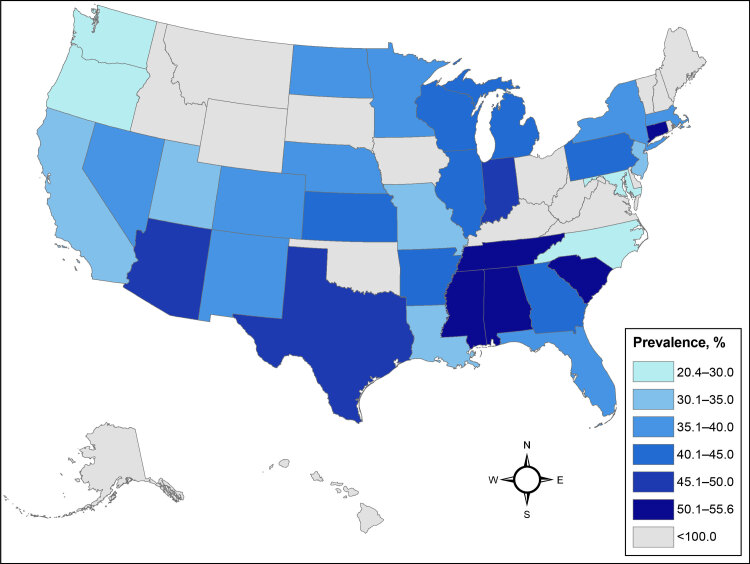
Prevalence of obesity in the US among the All of Us Research Program cohort with a calculated body mass index (kg/m^2^) of 30 or above, based on physical measurement data. Prevalence estimates were not calculated for states with fewer than 100 participants.

**Figure 2:**
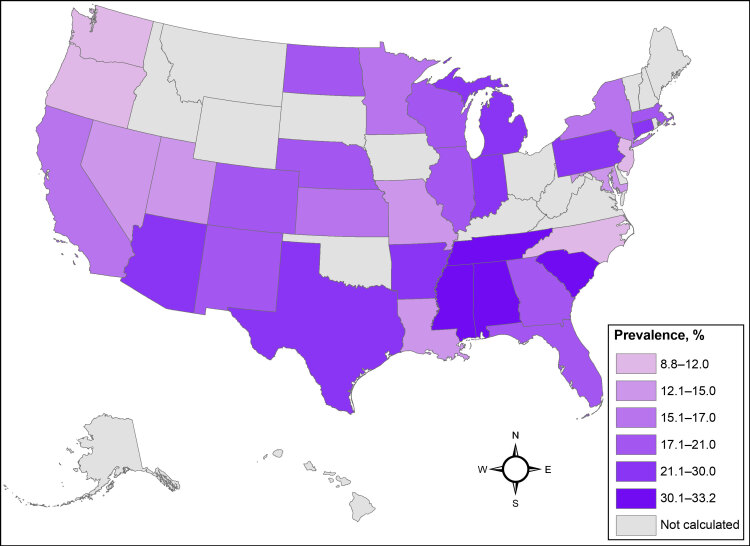
Prevalence of severe obesity in the All of Us Research Program, calculated BMI of 35 kg/m^2^ or above, based on physical measurement data. Prevalence estimates were not calculated for states with fewer than 100 participants.

**Obesity and severe obesity by geographic location and participant demographic characteristics**. Because of sample size limitations we calculated the prevalence of obesity and severe obesity nationwide for each contributing state, overall and stratified by binary sex assigned at birth. The prevalence estimates for obesity (BMI >30) and severe obesity (BMI >35) using All of Us physical measurements data were 41.5% (95% CI, 41.3%–41.7%) and 20.7% (95% CI, 20.6%–20.9%) with large variations across states (Table 2) (Figure 1) (Figure 2). Five states (Alabama, Connecticut, Mississippi, South Carolina, Tennessee) had overall obesity prevalence estimates greater than 50% (Table 2). Data from Connecticut were primarily collected from federally qualified health centers that serve as the All of Us hub in that state. Eight states (Alabama, Arkansas, Connecticut, Indiana, Mississippi, South Carolina, Tennessee, Texas) had severe obesity prevalence of 25% or greater (Table 2).

**Table 2:** Prevalence by State of Adult Obesity and Severe Obesity^a^ Using Physical Measurement Data, All of Us Research Program, May 2018–December 2020

Geographic Location	Total, N (%)	Male, n (%)	Female, n (%)	Overall Obesity (BMI^b^, ≥30), % (95% CI)			Severe Obesity (BMI^b^, ≥35), % (95% CI)		
				Overall	Male	Female	Overall	Male	Female
US Overall	244,504	95,944 (39)	148,560 (61)	41.5 (41.3–41.7)	35.7 (35.4–36.0)	45.2 (44.9–45.5)	20.7 (20.6–20.9)	14.7 (14.4–14.9)	24.6 (24.4–24.8)
Alabama	16,295 (6.7)	5,963 (37)	10,332 (63)	52.1 (51.3–52.9)	36.9 (35.7–38.2)	60.9 (59.9–61.8)	31.1 (30.4–31.8)	17.5 (16.6–18.5)	38.9 (38.0–39.9)
Arizona	31,354 (12.8)	12,452 (40)	18,902 (60)	46.2 (45.7–46.8)	39.8 (39.0–40.7)	50.5 (49.7–51.2)	24.5 (24.0–25.0)	18.1 (17.4–18.8)	28.7 (28.0–29.3)
Arkansas	152 (0.1)	58 (38)	94 (62)	44.1 (36.2–52.0)	37.9 (25.4–50.4)	47.9 (37.8–58.0)	25.0 (18.1–31.9)	13.8 (4.9–22.7)	31.9 (22.5–41.3)
California	34,262 (14.0)	13,021 (38)	21,241 (62)	34.9 (34.4–35.4)	32.5 (31.7–33.3)	36.4 (35.7–37.0)	15.5 (15.1–15.9)	11.8 (11.2–12.3)	17.8 (17.3–18.3)
Colorado	113 (0.05)	49 (43)	64 (57)	39.8 (30.8–48.8)	32.7 (19.5–45.8)	45.3 (33.1–57.5)	19.5 (12.2–26.8)	10.2 (1.7–18.7)	26.6 (15.7–37.4)
Connecticut	1,212 (0.5)	495 (41)	717 (59)	50.2 (47.4–53.0)	37.8 (33.5–42.0)	58.7 (55.1–62.3)	28.8 (26.2–31.3)	17.8 (14.4–21.1)	36.4 (32.9–39.9)
Florida	11,408 (4.7)	5,192 (46)	6,216 (54)	38.4 (37.5–39.3)	32.7 (31.4–34.0)	43.2 (42.0–44.4)	17.3 (16.6–18.0)	11.8 (11.0–12.7)	21.8 (20.8–22.8)
Georgia	5,472 (2.2)	2,518 (46)	2,954 (54)	40.1 (38.8–41.4)	31.7 (29.9–33.5)	47.2 (45.4–49.0)	19.4 (18.4–20.5)	11.6 (10.4–12.9)	26.0 (24.5–27.6)
Illinois	24,423 (10.0)	11,189 (46)	13,234 (54)	40.2 (39.6–40.9)	31.5 (30.7–32.4)	47.6 (46.8–48.5)	20.7 (20.2–21.2)	13.2 (12.6–13.8)	27.0 (26.2–27.7)
Indiana	113 (0.05)	49 (43)	64 (57)	46.9 (37.7–56.1)	38.8 (25.1–52.4)	53.1 (40.9–65.4)	25.7 (17.6–33.7)	16.3 (6.0–26.7)	32.8 (21.3–44.3)
Kansas	156 (0.1)	119 (76)	37 (24)	43.6 (35.8–51.4)	43.7 (34.8–52.6)	43.2 (27.3–59.2)	16.7 (10.8–22.5)	16.0 (9.4–22.5)	18.9 (6.3–31.5)
Louisiana	2,839 (1.2)	1,820 (64)	1,019 (36)	30.3 (28.6–32.0)	22.7 (20.8–24.7)	43.9 (40.8–46.9)	14.6 (13.3–15.9)	8.5 (7.2–9.7)	25.6 (22.9–28.3)
Maryland	104 (0.04)	43 (41)	61 (59)	25.0 (16.7–33.3)	16.3 (5.2–27.3)	31.1 (19.5–42.8)	13.5 (6.9–20.0)	4.7 (0.0–10.9)	19.7 (9.7–29.6)
Massachusetts	20,517 (8.4)	8,514 (41)	12,003 (59)	38.1 (37.5–38.8)	35.6 (34.5–36.6)	40.0 (39.1–40.9)	17.6 (17.1–18.1)	13.9 (13.2–14.7)	20.2 (19.5–20.9)
Michigan	11,997 (4.9)	3,956 (33)	8,041 (67)	44.7 (43.8–45.6)	38.8 (37.3–40.3)	47.6 (46.5–48.7)	24.0 (23.2–24.7)	16.9 (15.7–18.0)	27.5 (26.5–28.4)
Minnesota	822 (0.3)	284 (35)	538 (65)	36.9 (33.6–40.2)	40.1 (34.4–45.8)	35.1 (31.1–39.2)	16.1 (13.5–18.6)	15.8 (11.6–20.1)	16.2 (13.1–19.3)
Mississippi	3,058 (1.3)	1,217 (40)	1,841 (60)	55.4 (53.7–57.2)	37.2 (34.5–39.9)	67.5 (65.3–69.6)	33.2 (31.5–34.9)	16.4 (14.3–18.4)	44.3 (42.1–46.6)
Missouri	199 (0.1)	106 (53)	93 (47)	31.2 (24.7–37.6)	34.0 (24.9–43.0)	28.0 (18.8–37.1)	12.6 (8.0–17.2)	10.4 (4.6–16.2)	15.1 (7.8–22.3)
Nebraska	101 (0.04)	27 (27)	74 (73)	38.6 (29.1–48.1)	44.4 (25.7–63.2)	36.5 (25.5–47.5)	18.8 (11.2–26.4)	11.1 (0.0–23.0)	21.6 (12.2–31.0)
Nevada	168 (0.1)	67 (40)	101 (60)	36.3 (29.0–43.6)	41.8 (30.0–53.6)	32.7 (23.5–41.8)	14.9 (9.5–20.3)	14.9 (6.4–23.5)	14.9 (7.9–21.8)
New Jersey	240 (0.1)	91 (38)	149 (62)	30.4 (24.6–36.2)	31.9 (22.3–41.4)	29.5 (22.2–36.9)	10.0 (6.2–13.8)	12.1 (5.4–18.8)	8.7 (4.2–13.3)
New Mexico	221 (0.1)	106 (48)	115 (52)	38.0 (31.6–44.4)	33.0 (24.1–42.0)	42.6 (33.6–51.6)	18.1 (13.0–23.2)	15.1 (8.3–21.9)	20.9 (13.4–28.3)
New York	23,537 (9.6)	7,405 (31)	16,132 (69)	37.3 (36.7–37.9)	31.5 (30.4–32.6)	39.9 (39.2–40.7)	16.3 (15.8–16.8)	11.6 (10.9–12.3)	18.5 (17.9–19.1)
North Carolina	346 (0.1)	125 (36)	221 (64)	26.0 (21.4–30.6)	25.6 (17.9–33.3)	26.2 (20.4–32.0)	11.3 (7.9–14.6)	8.0 (3.2–12.8)	13.1 (8.7–17.6)
North Dakota	100 (0.04)	31 (31)	69 (69)	37.0 (27.5–46.5)	45.2 (27.6–62.7)	33.3 (22.2–44.5)	21.0 (13.0–29.0)	19.4 (5.4–33.3)	21.7 (12.0–31.5)
Oregon	113 (0.05)	45 (40)	68 (60)	20.4 (12.9–27.8)	20.0 (8.3–31.7)	20.6 (11.0–30.2)	8.8 (3.6–14.1)	11.1 (1.9–20.3)	7.4 (1.1–13.6)
Pennsylvania	23,803 (9.7)	7,741 (33)	16,062 (67)	43.2 (42.6–43.9)	41.2 (40.1–42.3)	44.2 (43.4–45.0)	21.7 (21.2–22.2)	17.4 (16.5–18.2)	23.8 (23.1–24.5)
South Carolina	1,494 (0.6)	377 (25)	1,117 (75)	55.6 (53.1–58.1)	38.2 (33.3–43.1)	61.5 (58.7–64.4)	32.2 (29.8–34.6)	18.6 (14.6–22.5)	36.8 (34.0–39.6)
Tennessee	1,336 (0.5)	478 (36)	858 (64)	54.1 (51.4–56.8)	44.6 (40.1–49.0)	59.4 (56.2–62.7)	32.9 (30.4–35.5)	24.7 (20.8–28.6)	37.5 (34.3–40.8)
Texas	8,302 (3.4)	3,003 (36)	5,299 (64)	49.9 (48.8–51.0)	44.8 (43.0–46.5)	52.8 (51.4–54.1)	26.5 (25.6–27.5)	20.6 (19.2–22.1)	29.9 (28.6–31.1)
Utah	128 (0.1)	51 (40)	77 (60)	33.6 (25.4–41.8)	27.5 (15.2–39.7)	37.7 (26.8–48.5)	14.8 (8.7–21.0)	9.8 (1.6–18.0)	18.2 (9.6–26.8)
Washington	426 (0.2)	180 (42)	246 (58)	26.1 (21.9–30.2)	22.2 (16.1–28.3)	28.9 (23.2–34.5)	10.1 (7.2–13.0)	4.4 (1.4–7.5)	14.2 (9.9–18.6)
Wisconsin	10,970 (4.5)	4,013 (37)	6,957 (63)	40.5 (39.6–41.4)	37.2 (35.7–38.7)	42.4 (41.3–43.6)	20.0 (19.2–20.7)	15.2 (14.1–16.3)	22.7 (21.7–23.7)
State information suppressed for privacy	8,723 (3.6)	5,159 (59)	3,564 (41)	37.7 (36.7–38.7)	38.9 (37.6–40.3)	36.0 (34.4–37.5)	16.0 (15.3–16.8)	14.6 (13.6–15.5)	18.2 (16.9–19.4)

Abbreviation: BMI, body mass index.

a Data on physical measurements of All of Us participants; includes data on participants with known age, binary sex, race, education, and from states with at least 100 participants. Values are percentage (95% CI).

b BMI = weight in kg ÷ height in m^2^.

Women in All of Us in each state had a higher prevalence of obesity than men except for 7 states: Kansas, Minnesota, Missouri, Nebraska, Nevada, New Jersey, and North Dakota (Table 2). Women had a higher prevalence of severe obesity than men in all states except Nevada, New Jersey, and Oregon. The prevalence of obesity and severe obesity differed by race and ethnicity, education, and age, qualitatively reflecting nationwide patterns seen in BRFSS data (Table 3) (1).

**Table 3:** Prevalence of Obesity and Severe Obesity^a^, Participants in the All of Us Research Program, by Demographic Characteristics, May 2018–December 2020

Demographic Characteristic	Total, N	Overall Obesity (BMI^b^ ≥30), n (Percentage) [95% CI]	Severe Obesity (BMI^b^ ≥35), n (Percentage) [95% CI]
**Race or ethnicity**			
Non-Hispanic White	120,519	45,127 (37.4) [37.3–37.6]	21,310 (17.7) [17.5–17.8]
Non-Hispanic Black	57,341	28,128 (49.1) [48.9–49.3]	16,104 (28.1) [27.9–28.3]
Hispanic	50,889	24,084 (47.3) [47.1–47.5]	11,387 (22.4) [22.2–22.5]
Other race or ethnicity^c^	15,755	4,050 (25.7) [25.5–25.9]	1,845 (11.7) [11.6–11.8]
**Education**			
Less than high school diploma or equivalent	28,709	13,189 (45.9) [45.7–46.1]	6,532 (22.8) [22.6–22.9]
High school diploma or equivalent to some college	118,906	56,363 (47.4) [47.2–47.6]	30,068 (25.3) [25.1–25.5]
College graduate	96,889	31,837 (32.9) [32.7–33.0]	14,046 (14.5) [14.4–14.6]
**Age, y**			
18–39	72,110	26,635 (36.9) [36.7–37.1]	14,486 (20.1) [19.9–20.2]
40–64	114,472	52,866 (46.2) [46.0–46.4]	27,369 (23.9) [23.7–24.1]
≥65	57,922	21,888 (37.8) [37.6–38.0]	8,791 (15.2) [15.0–15.3]

Abbreviation: BMI, body mass index.

a Physical measurement data among states with at least 100 participants.

b BMI = weight in kg ÷ height in m^2^.

c Participants for whom data are not reported separately because of small sample sizes. These include those responding none of these, Asian, more than one race or ethnicity, or another single race or ethnicity.

**Correlation between physical measurements and EHR data.** BMI data from physical measurements were highly correlated with BMI data from EHRs, with a Pearson correlation coefficient of 0.973 (95% CI, 0.972–0.973). Where data were available, the height and weight measurements from physical measurements and EHR data were similar and highly correlated with Pearson correlation coefficients >0.93 for height and >0.98 for weight in all subgroups and overall.

**Geographic variation and comparison to existing BRFSS data projections**. We compared state-level prevalence of obesity and severe obesity in the All of Us cohort with projections reported by Ward et al for the year 2020 calculated by using BRFSS survey data corrected for self-reporting bias (1). Seven states exhibited a 10% or greater absolute difference from state-level BRFSS projections for obesity prevalence (Connecticut, Louisiana, Maryland, Missouri, North Carolina, Oregon, Washington) and 4 states for severe obesity (Connecticut, Louisiana, Oregon, South Carolina). Larger variation in state-level prevalence estimates was significantly associated with smaller state sample sizes, with a Pearson correlation coefficient of −0.50 (95% CI, −0.72 to −0.19).

## Discussion

Our examination of the All of Us cohort data found a high prevalence of obesity (41.5%) and severe obesity (20.7%), which was consistent with national projections for the year 2020, as calculated from BRFSS data by Ward et al, projecting obesity and severe obesity prevalence at 42.0% and 19.4% respectively (1). We found geographic differences in obesity and severe obesity in the All of Us cohort, with the highest prevalence of each condition in states in the Southeast. Binary sex, race and ethnicity, and education patterns in obesity and severe obesity were also observed to be similar to national data (1). Our state-level results are congruent with those of Ward et al, with estimates differing by 10% in places that contributed small sample sizes to the analysis (1). Data from in-person physical measurements and clinical data from EHRs were tightly correlated, providing a measure of concordance validating these data sources.

Our findings suggest features of the All of Us cohort that may be relevant to promoting health equity and precision health in obesity research. Compared with White groups, non-Hispanic Black and Hispanic groups had a higher prevalence of obesity and severe obesity (12). Our analysis shows that the All of Us cohort is diverse with respect to race and ethnicity, age, education, and categories of body weight, which may enhance studies examining risks and associations in multiple population subgroups with social exposures that vary by geographic location and other factors. This is important because inclusion of diverse populations and a focus on addressing social inequities are key components of a precision population health framework for obesity prevention to provide tailored population health and prevention strategies (13,14).

Our study benefits from standardized measurements of weight and height from in-person physical measurements as opposed to self-reported measurements. Data from EHRs and physical measurements were closely correlated, providing some measure of construct validity in data collection. Future studies using the All of Us cohort may benefit from linkages with clinical EHR data, survey data, and biomeasures to better understand genomic, clinical, environmental, and social contributors to obesity and related conditions.

Our study had several limitations related to this early stage of analysis of All of Us data. Several states had few participants (<100), and we found great variability in the sample recruited by each site, which leads to variability in the precision of the state-level prevalence estimate. Additionally, All of Us is not designed as a representative geographic sample of the US (3). For example, states such as Connecticut predominantly recruited participants from federally qualified health centers, who may have different participant demographic characteristics than the state at large. Thus, prevalence estimates from All of Us are not expected to track estimates from surveys designed to produce representative population-based statistics. Our analysis did find similar obesity prevalence estimates in this analysis of All of Us data compared with studies designed to produce population-based estimates at the state level, which suggests that the All of Us cohort may provide the diversity in health status and geography needed to support investigations of risk factors that will advance the prevention and treatment of obesity. However, some racial and ethnic groups were not represented in sufficient numbers for large-scale analyses. All of Us continues to build community partnerships to increase accessibility to the program for diverse groups. Related to data availability, as of December 2020, the All of Us Researcher Workbench had EHR data for height and weight from 36% of the cohort included in our analysis. An important limitation is that EHR data differed by demographics and geographic region. Additional efforts will be important to ensure equitable availability of clinical data for subgroups who do not contribute data because of structural inequities, preferences, and other factors.

Our study’s ability to reproduce nationwide statistics was largely due to availability of physical measurements data obtained through All of Us. The ability to reproduce data may not be applicable to EHR data, which are not validated through medical record review, patient interviews, or similar processes that formed the basis of a sensitivity analysis for our study. Validation of EHR-based data elements and patient phenotypes may be important for future studies.

In summary, our demonstration project using existing methods for estimating obesity in the All of Us cohort shows parallels in obesity estimates found in data using national probability samples. Our analysis suggests 3 important points: 1) that All of Us has captured significant diversity within the US along the lines of binary sex, race and ethnicity, and age; 2) that the data show good internal consistency between physical measurements and EHR data and good external validity when compared with a second population-level study; and 3) that the data may have sufficient geographic spread to be useful for population-health studies and individual-level studies of contributors to obesity (1). As All of Us continues enrollment and adds data types (including genomics and biomarkers), additional studies are warranted to continue to monitor the applicability of the data to diverse populations.
